# *Trpc6* inactivation confers protection in a model of severe nephrosis in rats

**DOI:** 10.1007/s00109-018-1648-3

**Published:** 2018-05-22

**Authors:** Eun Young Kim, Parisa Yazdizadeh Shotorbani, Stuart E. Dryer

**Affiliations:** 1 Department of Biology and Biochemistry, University of Houston, Houston, TX USA; 2 Department of Medicine, Division of Nephrology, Baylor College of Medicine, Houston, TX USA

**Keywords:** Chronic kidney disease, TRPC6, FSGS, Glomerulosclerosis

## Abstract

**Abstract:**

Mutations in canonical transient receptor potential-6 (TRPC6) channels give rise to rare familial forms of focal and segmental glomerulosclerosis (FSGS). Here we examined a possible role for TRPC6 in the progression of chronic puromycin aminonucleoside (PAN) nephrosis in Sprague-Dawley rats, a classic model of acquired nephrotic syndromes. We used CRISPR/Cas9 technology to delete a 239-bp region within exon 2 of the *Trpc6* gene (*Trpc6*^del^ allele). *Trpc6*^del/del^ rats expressed detectable *Trpc6* transcripts missing exon 2, and TRPC6 proteins could be detected by immunoblot of renal cortex. However, the abundance of *Trpc6* transcripts and TRPC6 protein in renal cortex was much lower than in *Trpc6*^wt/wt^ littermates, and functional TRPC6 channels could not be detected in whole-cell recordings from glomerular cells cultured from *Trpc6*^del/del^ animals, possibly because of disruption of ankyrin repeats 1 and 2. During the chronic phase of PAN nephrosis, *Trpc6*^del/del^ rats had reduced urine albumin excretion, reduced serum cholesterol and triglycerides, and improved azotemia compared to wild-type *Trpc6*^wt/wt^ littermates. Glomerulosclerosis was severe during chronic PAN nephrosis in *Trpc6*^wt/wt^ rats but was markedly reduced in *Trpc6*^del/del^ littermates. *Trpc6*^del/del^ animals also had less severe tubulointerstitial fibrosis as assessed by several biochemical and histological analyses, as well as reduced foot process effacement and glomerular basement thickening compared to *Trpc6*^wtt/wt^ controls. None of the manipulations in this study affected the abundance of TRPC5 channels in renal cortex. TRPC3 was increased in PAN nephrosis and in *Trpc6*^del/del^ rats. These data support a role for TRPC6 channels in driving an acquired form of secondary FSGS.

**Key messages:**

- We examined aminonucleoside nephrosis in rats with wild type and inactivated TRPC6.
- TRPC6 channels were inactivated by CRISPR/Cas9 editing of the *Trpc6* gene.
- TRPC6 inactivation reduced albuminuria in the chronic but not the acute phase.
- TRPC6 inactivation reduced glomerulosclerosis and ultrastructural changes.
- TRPC6 inactivation also reduced interstitial changes and renal fibrosis.

**Electronic supplementary material:**

The online version of this article (10.1007/s00109-018-1648-3) contains supplementary material, which is available to authorized users.

## Introduction

Focal and segmental glomerulosclerosis (FSGS) occurs following a sufficient loss of podocytes [1, 2], often secondary to other pathological states [3]. A primary form of FSGS occurs as a result of circulating factors that drive glomerular dysfunction through poorly understood mechanisms [4, 5]. In addition, genetic studies have identified gain-of-function mutations in the *Trpc6* gene in patients with familial FSGS [6–10]. *Trpc6* encodes a widely expressed Ca^2+^-permeable channel (TRPC6) found in mesangial cells, podocytes, and other cells [11].

Familial FSGS associated with *Trpc6* mutations is rare [10], and it is not known if dysregulation of wild-type TRPC6 channels contributes to acquired FSGS. Patients with non-familial glomerular diseases, including primary FSGS, express TRPC6 at higher levels within glomeruli [12]. It is not known if this plays a role in driving the disease progression or, alternatively, if TRPC6 upregulation is a compensatory mechanism or a non-specific downstream marker of disease processes. We have recently shown that podocyte TRPC6 dysregulation can be driven by circulating factors present in patients with primary FSGS [13].

Previous studies have attempted to determine if TRPC6 contributes to kidney dysfunction and glomerulosclerosis using genetic manipulations of TRPC6 in mice [14, 15]. However, the manipulations that were used to produce FSGS-like lesions in those studies constrain conclusions that can be drawn since the kidney disease was transient and mild, even in *Trpc6*^+/+^ controls [14] or entailed genetic manipulations that cause artificial sustained TRPC6 activation [15, 16]. Beyond considerations of earlier experimental designs, there is value in addressing disease mechanisms in multiple species [17].

Here we have examined if TRPC6 channels play a role in the progression of chronic puromycin aminonucleoside (PAN) nephrosis in rats, an extensively studied model of severe secondary FSGS [1]. Because of its severity, this model more closely resembles patterns seen in human glomerular diseases than mouse models used to date. It does not require additional manipulations that artificially sustain TRPC6 activation. We also used CRISPR/Cas9 genome editing to delete a 239-bp region within exon 2 of *Trpc6*, which introduced numerous stop codons downstream of the deletion. Truncated *Trpc6* transcripts and TRPC6 proteins could still be detected, possibly as a result of posttranscriptional skipping of exon 2 [18, 19]. However, TRPC6 abundance was much lower in renal cortex of *Trpc6*^del/del^ rats, and we were unable to detect functional TRPC6 channels in glomerular cells cultured from those animals. Consequently, it was still possible to test the central hypothesis of this study.

## Material and methods

### Genome editing procedures

*Trpc6*^del/del^ rats were generated using CRISPR/Cas9 technology through a contract with a commercial vendor (Transposagen Biopharmaceuticals Inc.). The guide RNAs targeted a 239-bp domain in exon 2 of the rat *Trpc6* gene. The sequences of the two CRISPR guide RNAs used were as follows:

2.1 GGAAGAAGGTTGGCTAATCG
2.2 GGAAAACTTGTCTCGAGTTG

These guide RNAs were designed to be specific for *Trpc6* and do not target genes encoding closely related channel proteins (see Supplemental Material). It is important to note that we cannot entirely exclude that they may have off-target effects elsewhere. Littermates bred from *Trpc6*^wt/del^ heterozygotes were genotyped using PCR primers that span the deleted region of the *Trpc6* gene (primer sequences are in Supplemental Material Table 1). RT-PCR analyses of total RNA extracted from renal cortex of *Trpc6*^wt/wt^ and *Trpc6*^del/del^ rats was carried out using primers designed to span various portions of the *Trpc6* transcript, including exons 1–2, exons 2–4, exons 4–8, and exons 8–13. We also used a primer pair that spanned a short region on either side of the deleted region (exon 1–Δ2) and a primer pair to detect *Actb*.

### Glomerular cell isolation and electrophysiology

Decapsulated glomeruli were isolated from *Trpc6*^wt/wt^ and *Trpc6*^del/del^ rats [20]. Epithelial cells that grew out of the glomeruli after 2 days were re-plated onto collagen-coated glass coverslips and used for electrophysiology as described previously [13, 21]. Data are from cells isolated from at least three animals in each group. Recordings were made with an Axopatch 1D amplifier (Molecular Devices) and analyzed using PClamp™ v 10 software (Molecular Devices). The bath solution contained 150 mM NaCl, 5.4 mM CsCl, 0.8 mM MgCl_2_, 5.4 mM CaCl_2_, and 10 mM HEPES, pH 7.4 at 1.5 ml/min. Pipette solutions in all experiments contained 10 mM NaCl, 125 mM CsCl, 6.2 mM MgCl_2_, 10 mM HEPES, and 10 mm EGTA, pH 7.2. Briefly, after making intracellular contact, cells were held at − 40 mV. Currents were periodically monitored during application of voltage ramps (− 80 to + 80 mV over 2.5 s), before and after application of 100 μM ATP by gravity-fed superfusion. We have previously shown that ATP activates TRPC6 channels in podocytes through actions on G protein-coupled P2Y receptors [21]. ATP-evoked currents occurred within 60–90 s after switching bath solutions, corresponding to the dead time of the perfusion system and chamber. ATP-evoked currents were blocked by 100 nM SAR-7334 (MedChem Express), a highly specific inhibitor of TRPC6 [22]. Currents were quantified at + 80 mV and are presented as the fold change in presence of ATP over baseline. We also analyzed the percentage of cells with discernible increases in current evoked by ATP in the two groups.

### Chronic PAN nephrosis

These protocols were approved by the University of Houston Institutional Animal Care and Use Committee following NIH guidelines. *Trpc6*^wt/wt^ rats and *Trpc6*^del/del^ littermates (100–150 g) were given two injections of PAN (Sigma-Aldrich) dissolved in 0.9% sterile saline. A first injection of 200 mg/kg i.p. was followed by a second injection of 100 mg/kg i.p. administered 30 days later. Controls received the saline vehicle at the same times. Urine albumin was measured using a commercial ELISA assay (Exocell Inc.). Mean arterial blood pressure was measured between by tail-cuff plethysmography (CODA 6, Kent Scientific). At 30 days after the second injection, blood was sampled and animals were sacrificed by CO_2_ inhalation followed by cervical dislocation and the kidneys and heart were excised and weighed. A portion of renal cortex of one kidney was removed and reserved for biochemical, histological, and ultrastructural analysis.

### Immunoblot analysis

These methods were described previously [13]. Renal cortex was homogenized in M-PER™ mammalian protein extraction buffer (Thermo Fisher Scientific) and sonicated on ice. The homogenates were subjected to centrifugation at 13,000 rpm for 30 min. Protein concentrations of the supernatants were determined with Bradford reagent (Bio-Rad). Rabbit antibodies against TRPC6 (ACC-017) and TRPC3 (ACC-016) were from Alomone Labs. The TRPC6 antibody targets motifs encoded by exon 1 located in the amino-terminal and upstream of the deletion in the *Trpc6*^del^ allele. We also used a polyclonal TRPC6 antibody from Booster Biological Technology that targets residues 248–264 in the rat channel, downstream of the deletion. A mouse monoclonal antibody against TRPC5 was from NeuroMAB. We also used a rabbit polyclonal antibody against TRPC5 from Alomone (ACC-020). All TRPC antibodies were used at a dilution of 1:1000. A mouse monoclonal antibody (clone 1A4) against α-smooth muscle actin (SMA) was from Sigma-Aldrich.

### Histology and ultrastructure

Portions of kidney were immersion-fixed, embedded in paraffin, and 3-μm serial sections staining by periodic acid-Schiff’s (PAS) and Masson’s trichrome methods and by immunohistochemistry. All pathological specimens were evaluated by an observer blind to the nature of the treatment group. For each animal, the glomerular score (GS) in PAS-stained sections was graded using a scale of 0–4: 0 was assigned to normal glomeruli, 1 denoted glomeruli with mesangial expansion, 2 denoted glomeruli in which sclerosis encompassed less than 50% of the glomerulus, 3 denoted glomeruli with lesions encompassing 50–75% of the glomerulus, and 4 denoted glomeruli with lesions encompassing more than 75% of the glomerulus or fully collapsed glomeruli. GS was evaluated in 25–50 glomeruli and averaged to obtain a mean value for each animal. Statistical analysis was carried out on the mean values from each group of animals, with *N* = 6 rats per group. Immunohistochemistry for the rat macrophage marker CD68 was carried out by ABC method using a monoclonal primary antibody against rat CD68 (ED-1) from Abcam (ab31630) at a dilution of 1:200. The average number of stained cells per glomerulus for each animal was determined by an observer blind to the treatment group. A portion of renal cortex was fixed by immersion in 3% glutaraldehyde plus 3% paraformaldehyde in 0.1 M sodium cacodylate buffer pH 7.3 for ultrastructural analysis as described in detail previously [23]. The thickness of the glomerular basement membrane was determined from these images. Podocyte foot process width (FPW) was calculated as FPW = π/4 × ∑GBM length / ∑foot process where ∑GBM length is the total GBM length measured in each picture and ∑foot process is the total number of foot processes counted in each picture. The term π/4 corrects for random variation in the angle of section relative to the long axis of the podocyte [24].

### Statistical analyses

All statistical analyses were carried out using public-access computational tools (http://www.vassarstats.net) with *P* < 0.05 regarded as significant. Immunoblot assays were performed in triplicate and analyzed by densitometry. The data are presented as fold changes relative to the lowest value observed in a control group and are presented as mean ± SD. They were analyzed by Student’s unpaired or Bonferroni’s *t* test. Electrophysiological data were analyzed by Mann-Whitney *U* test and by Fisher’s exact test. Data on 24-h urine albumin excretion and other quantitative measures of renal and metabolic status are presented as mean ± SEM and as scatter plots from *N* = 6 rats per group. Data were analyzed by two-way ANOVA followed by Tukey’s honest significant difference post hoc test The two independent variables were genotype (*Trpc6*^wt/wt^ vs. *Trpc6*^del/del^) and drug treatment (PAN vs. saline vehicle). A statistically positive result was inferred when *F* values for the interaction between drug effects and genotype indicated *P* < 0.05.

## Results

### Effects of the exon 2 deletion on renal cortical TRPC6 expression and function in glomerular cells

**Fig. 1:**
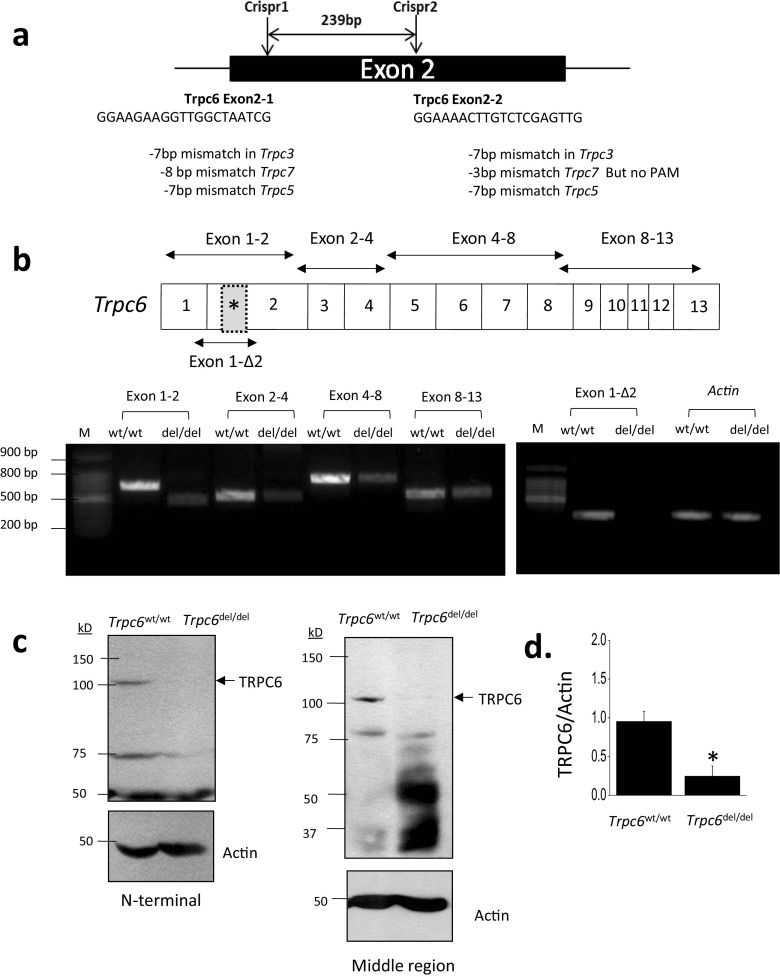
Generation of the *Trpc6*^del^ allele and its expression in *Trpc6*^del/del^ and *Trpc6*^wt/wt^ rats. **a** Schematic showing CRISPR/Cas9 strategy used to generate rats with a 239-bp deletion in the second exon of *Trpc6*. We initially expected this deletion to result in numerous frameshift mutations downstream of the deletion. The sequences of guide RNAs used to generate the animals are also shown. **b** Conventional RT-PCR of *Trpc6* transcripts in renal cortex using primer pairs that span different portions of the transcript. All of the primer pairs produced robust signal from renal cortex of *Trpc6*^wt/wt^ rats, but signal from all primer pairs was reduced in *Trpc6*^del/del^ littermates and was missing using the primer pair (1-Δ2) that targets residues very close to the deleted region. **c** Example of immunoblot analysis of TRPC6 in renal cortical extracts from a *Trpc6*^wt/wt^ and a *Trpc6*^del/del^ rat as indicated. The blot to the left showed signal obtained using an antibody targeting motifs upstream of the deletion, and the blot to the right was obtained using a different antibody targeting a sequence downstream of the deletion. Signal from *Trpc6*^wt/wt^ rats was easy to see but was extremely faint in the *Trpc6*^del/del^ animals. **d** Densitometric analysis of the signal obtained using the N-terminal antibody with *N* = 6 animals per group

**Fig. 2:**
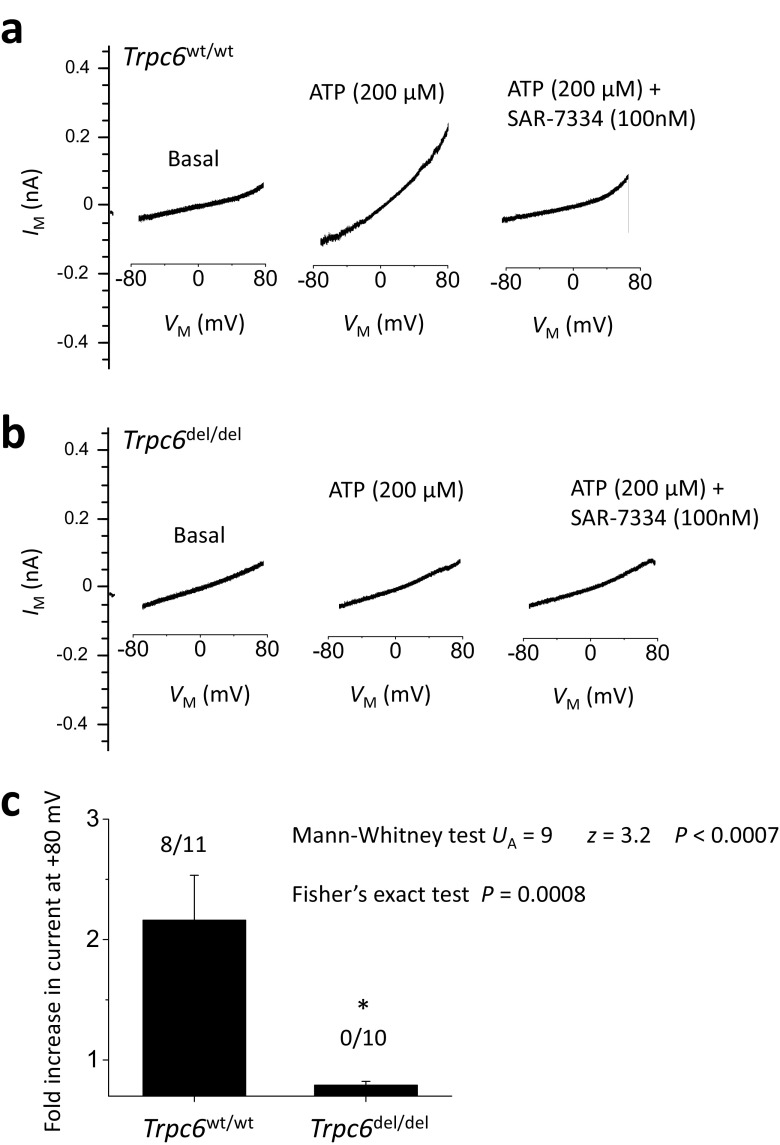
Whole-cell recordings from glomerular cells cultured from *Trpc6*^wt/wt^ and *Trpc6*^del/del^ rats. Representative traces of recordings showing currents before and after application of 100 μM ATP. Note increase in current in cells from *Trpc6*^wt/wt^ rats, and blockade of this currents by 100 nm SAR-7334, a selective blocker of TRPC6 (**a**). Note also that cells cultured from *Trpc6*^del/del^ rats did not respond to ATP (**b**). Bar graph in (**c**) shows mean fold increase in current evoked by ATP relative to baseline in cells from *Trpc6*^wt/wt^ and *Trpc6*^del/del^ rats. Error bars represent SEM. Numbers above bars indicate number of cells that respond to ATP over the total number of cells recorded. Data were highly statistically significant by Mann-Whitney *U* test and Fisher’s exact test for proportions

The strategy used to generate *Trpc6* exon 2 deletions in rats is summarized in Fig. 1a. The deletion of a 239-bp region within the *Trpc6* gene was confirmed by genomic sequencing and was readily seen in PCR analyses of genomic DNA used to genotype the animals. This deletion introduced numerous premature stop codons downstream of the deletion. However, we were able to detect *Trpc6* transcripts in renal cortex by RT-PCR using four different primer pairs, although these transcripts were present at substantially lower levels in *Trpc6*^del/del^ rats compared to *Trpc6*^wt/wt^ littermates (Fig. 1b). By contrast, when we used a primer pair that targeted regions of the transcript very close to the deleted region within exon 2 (exon 1Δ2), we obtained signals of the appropriate size in *Trpc6*^wt/wt^ rats but not in *Trpc6*^del/del^ littermates (Fig. 1b). We also observed TRPC6 subunits in immunoblot analysis of renal cortex of *Trpc6*^wt/wt^ and *Trpc6*^del/del^ rats, although the signal from *Trpc6*^del/del^ rats was very faint (Fig. 1c, d). This pattern was seen with two different commercial polyclonal antibodies that target different epitopes within the TRPC6 channel. Thus, *Trpc6*^del/del^ animals are able to express very low levels of TRPC6 subunits, possibly by splicing out the exon containing premature stop codons, a phenomenon reported by other investigators studying other genes modified by CRISPR/Cas9 methods [18, 19]. It is clear from the expression levels of transcripts and proteins that the *Trpc6*^del^ is a strongly hypomorphic allele. We confirmed this using whole-cell recordings of glomerular cells cultured from *Trpc6*^wt/wt^ and *Trpc6*^del/del^ animals (Fig. 2). We have previously shown that ATP activates TRPC6 channels in podocytes by activation of P2Y receptors [21]. Here we observed that 100 μM ATP caused robust activation of cationic currents in 8 out of 11 cells, in total cultured from three different *Trpc6*^wt/wt^ animals, and cells from all three animals responded to ATP. As we observed previously [21], those currents peaked within 60–90 s after the onset of gravity-fed perfusion with external saline containing ATP. The ATP-evoked currents were blocked completely by subsequent application of 100 nM SAR-7334, a selective inhibitor of TRPC6 [22] (Fig. 2a). By contrast, 100 μM ATP did not activate cationic currents in any of ten cells examined from three different *Trpc6*^del/del^ rats (*P* < 0.0007 by Mann-Whitney *U* test analysis of fold increases in current and *P* < 0.0008 by Fisher’s exact test of the numbers of cells with a discernible response).

### Urine albumin excretion and renal function

**Fig. 3:**
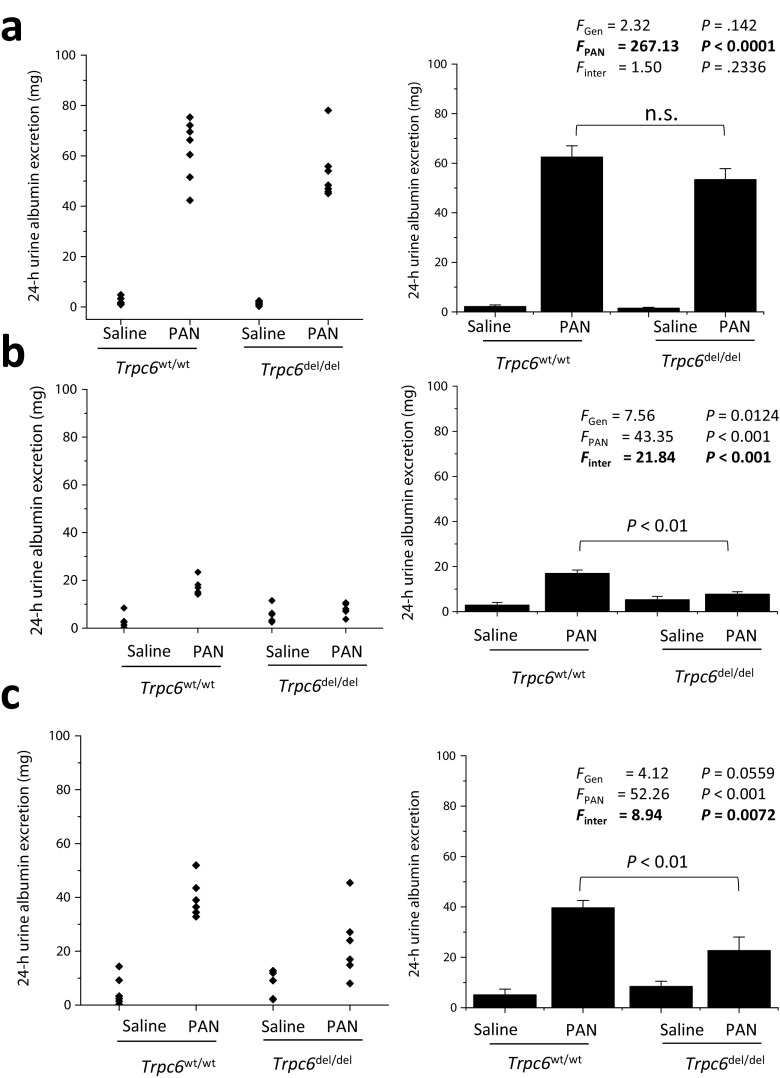
Reduced albuminuria in chronic PAN nephrosis in *Trpc6*^del/del^ rats compared to *Trpc6*^wt/wt^ littermates. **a** Urine albumin excretion (over 24 h) in rats 10 days after an initial PAN or saline injection, as indicated. Scatter graph on left shows urine albumin excretion from each animal in each group. Bar graph to the right shows mean ± SEM for the six animals in each group. Two-way ANOVA indicates marked effect of PAN on albumin excretion, but there was no difference in this effect between *Trpc6*^wt/wt^ and *Trpc6*^del/del^ rats, and no interaction between the effects of genotype and drug treatment. **b** In the same animals, 30 days after the first PAN injection, there is now a statistically robust difference in 24-h urine albumin excretion between *Trpc6*^wt/wt^ and *Trpc6*^del/del^ animals that received revealed by significant interaction effect between genotype and drug treatment and by Tukey’s honest significant difference post hoc test. At this stage, *Trpc6*^del/del^ animals appear to be completely protected from PAN-evoked albuminuria. A few days after these urine samples were collected, a second injection of PAN or saline was given to these rats. **c** Urine albumin excretion in the same animals, 30 days after the second PAN or saline injection (and 60 days after the first one). Urine albumin excretion was increased in PAN-treated animals. There is marked protection from PAN effects in *Trpc6*^del/del^ animals. Two-way ANOVA indicates statistically robust interaction between effects of PAN and genotype, and the post hoc test indicates marked difference between *Trpc6*^wt/wt^ and *Trpc6*^del/del^ rats that received PAN

PAN-evoked nephrosis in rats occurs in two phases [1, 25, 26]. An acute injury phase characterized by severe proteinuria occurs within days after a single PAN injection. At that time, no lesions can be seen by light microscopy. This recovers over the next 2–3 weeks but is followed by a sustained re-emergence of albuminuria that, while less severe, is accompanied by glomerulosclerosis [1, 25, 26]. The chronic phase of this model is relevant to human nephrotic syndromes. We observed no difference in 24-h urine albumin excretion in PAN-treated *Trpc6*^wt/wt^ and *Trpc6*^del/del^ rats during the acute phase, measured 10 days after the initial PAN injection (Fig. 3a). At that time, all PAN-treated animals exhibited severe proteinuria > 40 mg/24 h. There was no difference in albumin excretion between *Trpc6*^wt/wt^ and *Trpc6*^del/del^ rats that received saline. Two-way ANOVA revealed a robust effect of PAN, but no statistically significant interaction between the effects of genotype and PAN treatment at that stage (*P* = 0.2336).

A difference in the amount of 24-h urine albumin excretion in PAN-treated *Trpc6*^wt/wt^ and *Trpc6*^del/del^ rats was observed 30 days after the first PAN injection (Fig. 3b). At that time, PAN-treated *Trpc6*^wt/wt^ rats had a significant increase in 24-h urine albumin excretion compared to saline-treated controls (albeit less than in the acute phase), whereas there was no difference between PAN-treated *Trpc6*^del/del^ animals and saline-treated controls. There was a statistically significant interaction between the effects of genotype and PAN treatment (*P* < 0.001) on urine albumin excretion. At that time, the rats were given a second injection of PAN or saline. The protective effect in *Trpc6*^del/del^ animals was still observed more than 30 days after the *second* booster injection (which is 60 days into the disease process), and two-way ANOVA again revealed a robustly significant interaction effect between effects of genotype and PAN treatment on albumin excretion (*P* < 0.001), indicating a partial protective effect of exon 2 deletion (Fig. 3c). Albuminuria at the 60-day time point was not correlated with the degree of proteinuria seen at 10 days (see Supplemental Fig. 1). There was an increase in kidney weight/body weight ratio in PAN-treated rats compared to saline-treated controls, but this effect was not changed by *Trpc6* knockout (see Supplemental Figs. 2 and 3). PAN treatment and *Trpc6* exon 2 deletion had no effect on heart weight or mean arterial pressure (Supplemental Fig. 3).

**Fig. 4:**
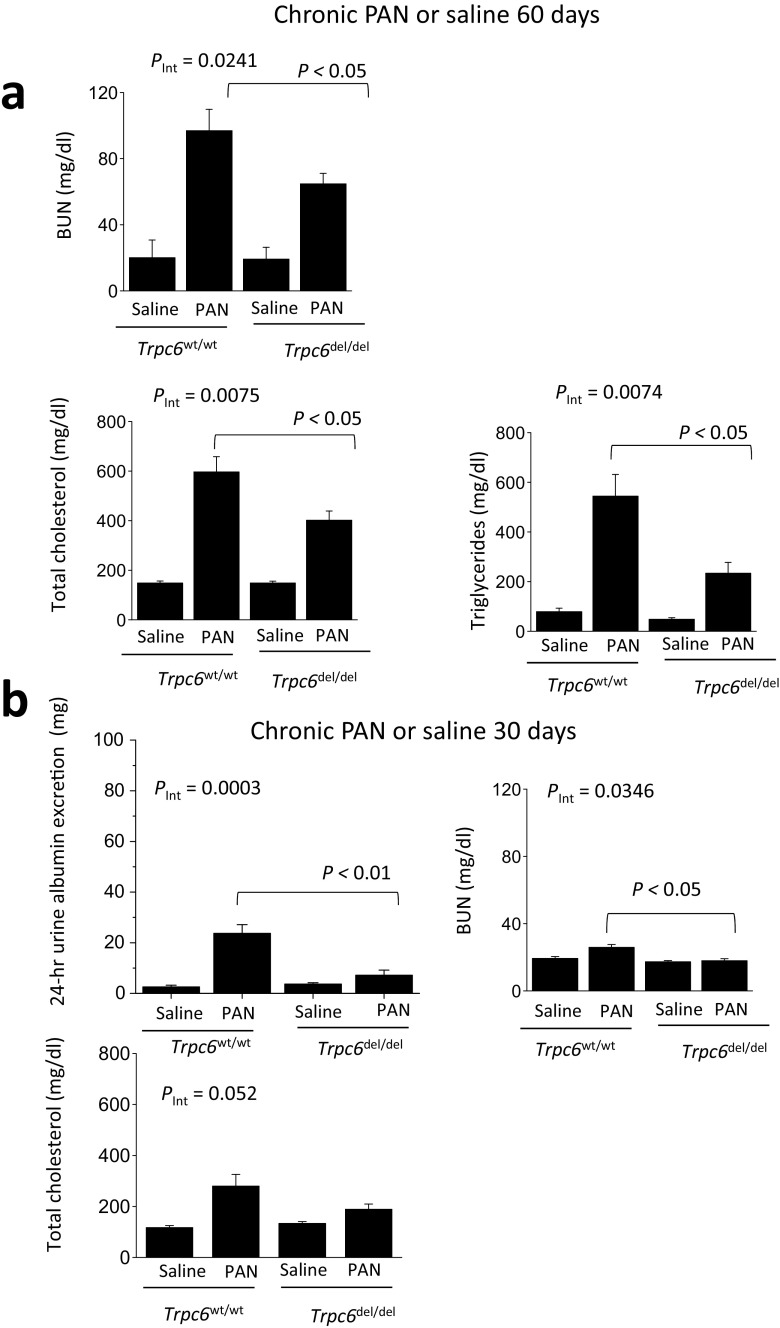
*Trpc6*^del/del^ rats are protected against changes in blood chemistry seen in chronic PAN nephrosis. **a** In the same animals shown in Fig. 3 immediately prior to sacrifice, we observed significant reductions in blood urea nitrogen (BUN), total serum cholesterol, and total serum triglycerides in PAN-treated *Trpc6*^del/del^ animals compared to *Trpc6*^wt/wt^ littermate controls. Two-way ANOVA showed significant interaction between genotype and drug treatment for all three measures, as well as significant differences between *Trpc6*^wt/wt^ and *Trpc6*^del/del^ in PAN-treated groups by post hoc test. As with albumin excretion, the protection was not complete. **b** In a second set of animals (*N* = 6 per group), we observed increased 24-h albumin excretion and BUN in PAN-treated *Trpc6*^wt/wt^ rats but not in *Trpc6*^del/del^ animals compared to saline-treated controls. Two-way ANOVA showed significant interaction between genotype and drug treatment for both measures, as well as significant differences between *Trpc6*^wt/wt^ and *Trpc6*^del/del^ in PAN-treated groups by post hoc test. At this earlier time point in PAN nephrosis, protective effect of TRPC6 knockout is essentially complete

The protective effect of *Trpc6*^del^ was observed with several other measurements. PAN-treated rats had increased blood urea nitrogen (BUN) compared to saline-treated controls, measured 60 days after the initial injection. This was less severe in *Trpc6*^del/del^ rats compared to *Trpc6*^wt/wt^ littermates, and two-way ANOVA revealed a statistically significant interaction between genotype and PAN treatment on BUN (*P* < 0.05) (Fig. 4a). Chronic PAN in *Trpc6*^wt/wt^ animals caused increases in serum total cholesterol and triglycerides (Fig. 4a), but this was less severe in *Trpc6*^del/del^ littermates (*P* < 0.05). In a separate experiment with six rats in each group, we measured 24-h urine albumin excretion, BUN, and serum lipids 30 days after injection of PAN or saline (Fig. 4b). In these rats (*N* = 6 per group), we again saw albuminuria and increased BUN in PAN-treated *Trpc6*^wt/wt^ rats but not in PAN-treated *Trpc6*^del/del^ rats, and the protective effect at 30 days was essentially complete (*P* < 0.05) (Fig. 4b).

### Histology and ultrastructural analysis

**Fig. 5:**
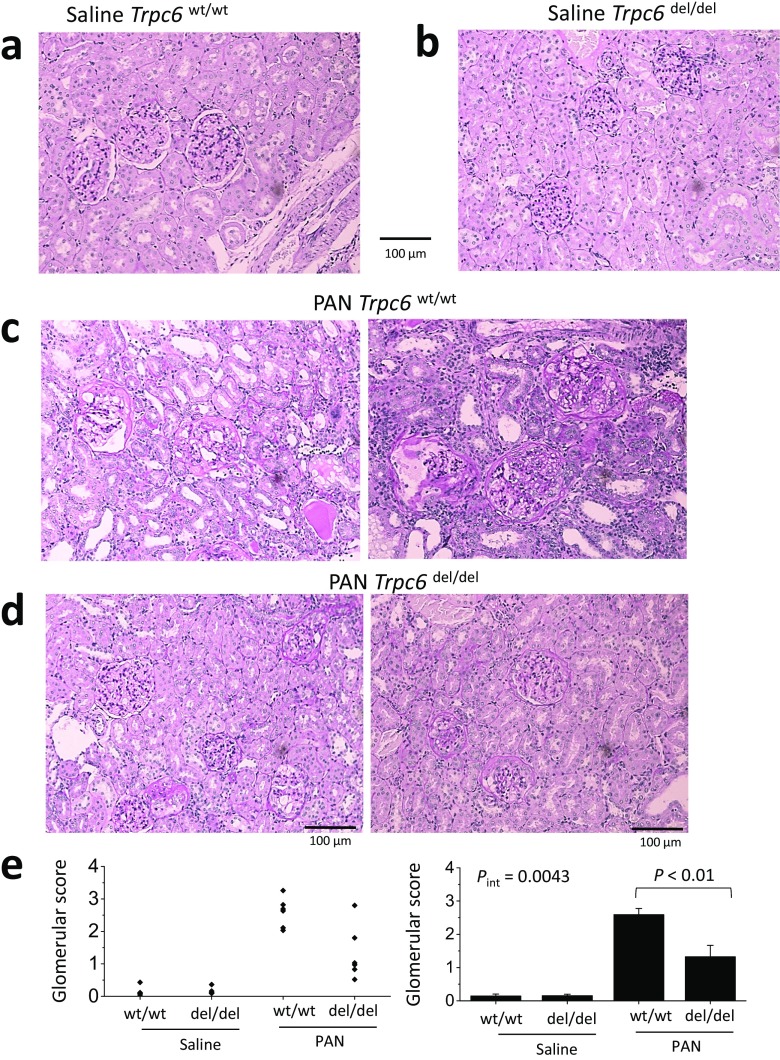
Histological analysis showing protective effect of *Trpc6* exon 2 deletion during chronic PAN nephrosis. PAS-stained sections were prepared after the animals in Fig. 3 were sacrificed. The deletion has no effect on renal histology in saline-treated rats (**a**, **b**). By contrast, there is severe kidney disease in *Trpc6*^wt/wt^ rats that received chronic PAN (**c**). Nearly all of the glomeruli in these animals showed at least some glomerulosclerosis and many were completely collapsed. There were also frequent protein casts within tubules, as well as tubular atrophy and marked interstitial hypercellularity. **d** There were still indications of kidney disease in *Trpc6*^del/del^ rats that received chronic PAN, but they were markedly less severe. **e** Mean glomerular score (GS) calculated in a blind manner from PAS-stained sections from each group, with scatter plot showing results from individual animals (left) and bar graph showing group mean ± SEM (right). A GS of 0 was assigned to normal glomeruli, one was assigned to glomeruli with mesangial expansion, two was assigned to glomeruli in which sclerosis encompassed less than 50% of the glomerulus, three was assigned to glomeruli with lesions that encompassed 50–75% of the glomerulus, and four was assigned to glomeruli with lesions encompassing more than 75% of the glomerulus, including fully collapsed glomeruli. For each animal, GS was evaluated in 25–50 glomeruli and averaged to obtain a mean value for that animal. Statistical analysis was carried out on the mean values from each group of animals, with *N* = 6 rats in each of the four groups. We observed reduced glomerulosclerosis in five out of six *Trpc6*^del/del^ rats. This effect was significant by two-way ANOVA and post hoc test

**Fig. 6:**
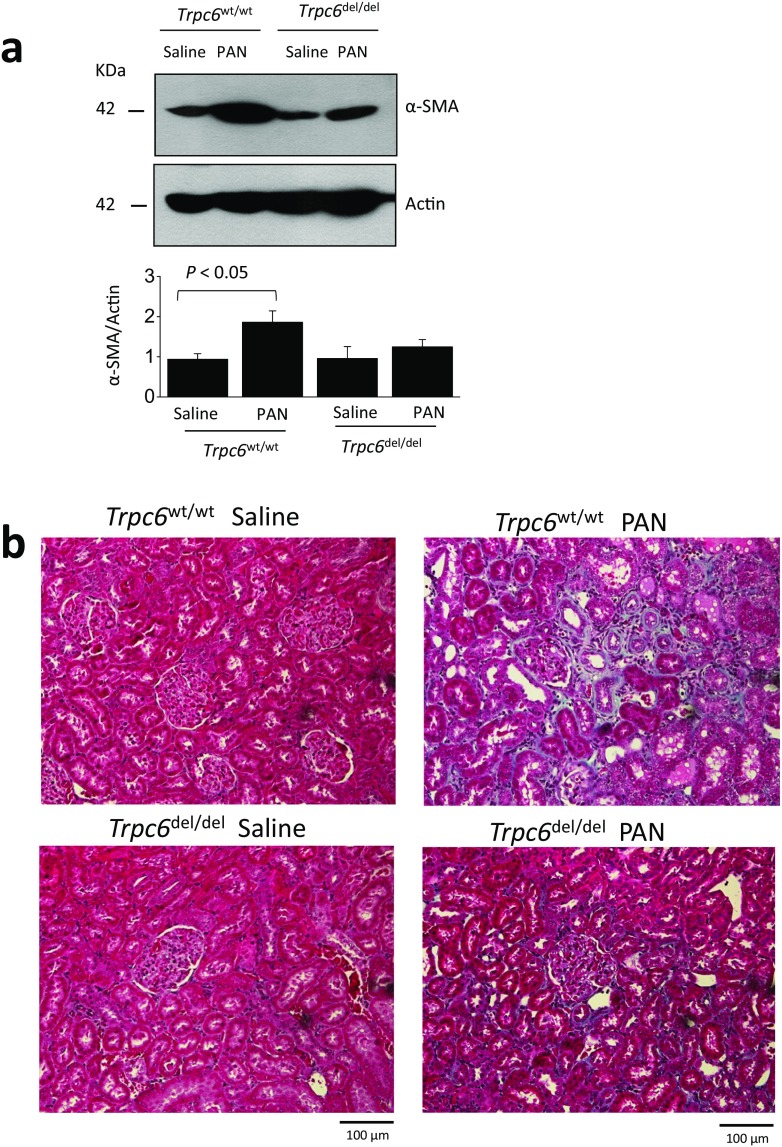
Kidney fibrosis in chronic PAN nephropathy is reduced in *Trpc6*^del/del^ rats. **a** Immunoblot analysis of α-SMA abundance of renal cortex indicates that fibrosis in PAN-treated *Trpc6*^wt/wt^ rats is more severe than in *Trpc6*^del/del^. **b** Masson’s trichrome staining showing that fibrosis extends to tubulointerstitial areas during chronic PAN nephrosis

**Fig. 7:**
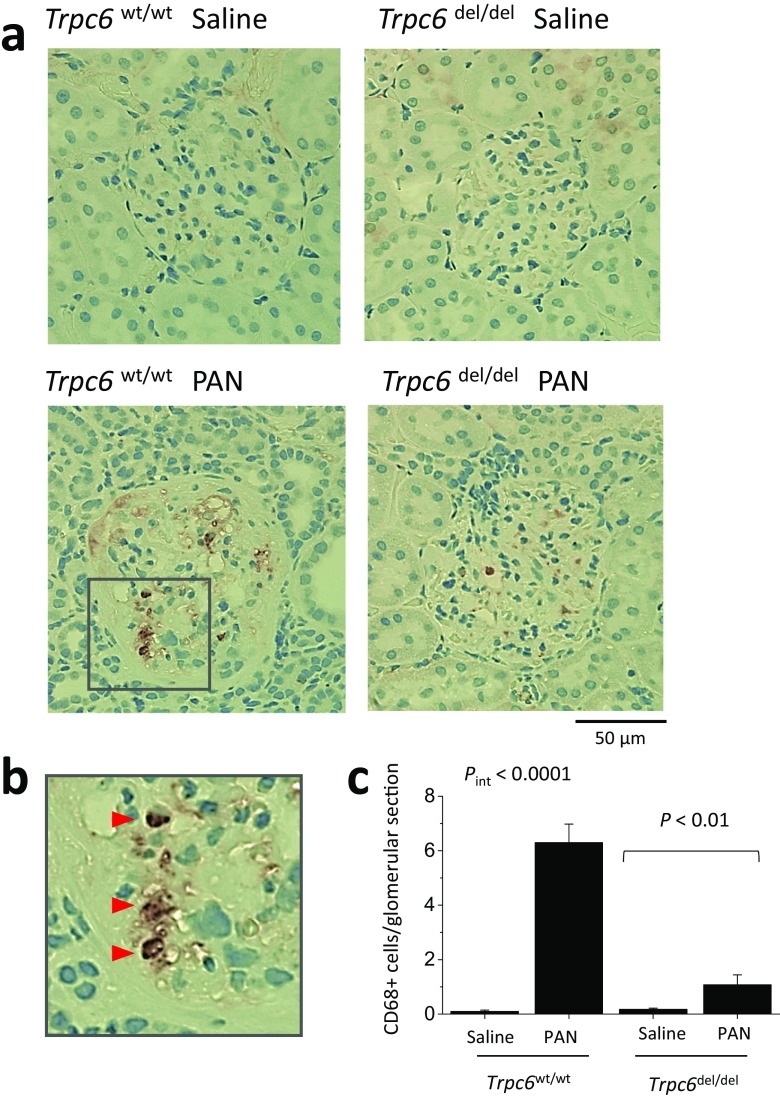
*Trpc6* exon 2 deletion reduces infiltration of CD68-expressing cells into glomeruli of PAN-treated animals. **a** Examples of immunohistochemistry using the monoclonal antibody ED-1, which is directed against CD68, a marker for macrophages and monocytes of myeloid lineage. Inset in lower right panel is shown at higher magnification in **b**. **c** Mean ± SEM of the average number of ED-1 stained cells per glomerular cross section in each animal (*N* = 6 rats per group). It should be noted however that we did not make serial sections through entire glomeruli. Cells were counted by an observer blind to the nature of the treatment groups

Animals were sacrificed after completion of the 60-day protocol. Histology assessed by PAS staining was normal in saline-treated *Trpc6*^wt/wt^ and *Trpc6*^del/del^ rats (Fig. 5a, b). However, there was marked glomerular scarring and many collapsed glomeruli in all of the PAN-treated *Trpc6*^wt/wt^ rats (Fig. 5c). Indeed, virtually, every glomerulus in PAN-treated *Trpc6*^wt/wt^ animals exhibited some degree of sclerosis. There was also extensive tubular atrophy, and many tubular protein casts, along with interstitial hypercellularity and fibrosis. The pathology was quantitatively and qualitatively less severe in PAN-treated *Trpc6*^del/del^ rats (Fig. 5d, e). It was possible to find many unaffected or mildly affected glomeruli in *Trpc6*^del/del^ rats. There were indications of tubulointerstitial disease, especially hypercellularity, but hyalinization in tubules was rare in PAN-treated *Trpc6*^del/del^ rats. A quantitative analysis of glomerulosclerosis in this experiment indicated a significant protective effect of exon 2 deletion (Fig. 5e, right) (*P* < 0.01 by two-way ANOVA). *Trpc6*^del/del^ rats also had reduced interstitial fibrosis. We detected substantially more α-smooth muscle actin (SMA) in the renal cortex of PAN-treated rats than in saline-treated controls. This increase was less severe in *Trpc6*^del/del^ rats compared to *Trpc6*^wt/wt^ littermates (Fig. 6a). This could also be seen in Masson’s trichrome staining (Fig. 6). We observed fewer cells expressing the monocyte/macrophage marker CD68 within the glomeruli of PAN-treated *Trpc6*^del/del^ rats compared to *Trpc6*^wt/wt^ rats (Fig. 7).

**Fig. 8:**
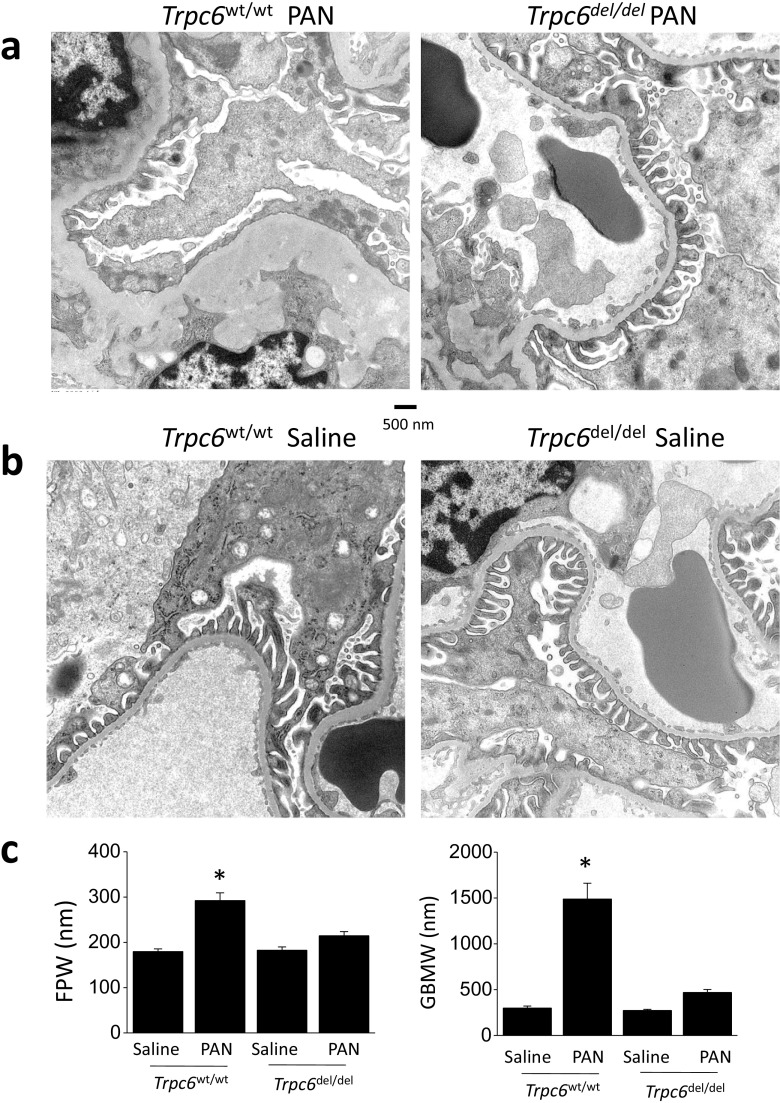
*Trpc6* exon 2 deletion reduces ultrastructural changes evoked by chronic PAN. These transmission electron micrographs were prepared from the same animals of Fig. 3 after sacrifice. **a** In *Trpc6*^wt/wt^ animals, chronic PAN nephrosis was associated with extensive foot process effacement and marked thickening and disruption and thickening of glomerular basement membrane (left). Many capillary loops no longer had discernible foot processes. In *Trpc6*^del/del^ rats, we still observed some effacement and thickening of foot processes. However, ultrastructure was substantially closer to normal (right). **b** *Trpc6* exon 2 deletion has no effect on glomerular ultrastructure in saline-treated rats. **c** Quantification of the effects of exon 2 deletion on mean foot process width (FPW) and GBM thickness (GBMW) in saline- and PAN-treated animals

Glomerular ultrastructure was indistinguishable in saline-treated *Trpc6*^wt/wt^ and *Trpc6*^del/del^ rats (Fig. 8). However, ultrastructure was more disrupted in PAN-treated *Trpc6*^wt/wt^ animals than *Trpc6*^del/del^ littermates, with more extensive foot process effacement and glomerular basement membrane (GBM) thickening. The differences in FPW and GBM thickening in PAN-treated *Trpc6*^wt/wt^ and *Trpc6*^del/del^ animals were quantitatively significant (Fig. 8; see also Supplemental Fig. 4).

## Discussion

TRPC6 channels have been implicated in familial forms of TRPC6, and it has been suggested that these channels also play a role in acquired nephrotic syndromes [12, 13]. The hypothesis that TRPC6 channels play a role in the progression of acquired FSGS predicts that pathology in chronic PAN nephrosis should be reduced when functional TRPC6 channels are not present. We tested this prediction in *Trpc6*^del/del^ Sprague-Dawley rats and their littermate *Trpc6*^wt/wt^ controls generated by CRISPR/Cas9 technology.

The *Trpc6*^del^ allele generated in this study is present at very low levels at both the mRNA and protein level *Trpc6*^del/del^ rats, and we were unable to detect functional TRPC6 channels in glomerular cells from those animals, in marked contrast to *Trpc6*^wt/wt^ controls. Nevertheless, the fact that TRPC6 subunits could be detected using two different antibodies, in spite of the fact that the deletion was predicted to induce a shift in the reading frame, indicates precautions that should be taken when using CRISPR/Cas9 technology. Indeed, conclusions of this study must also be tempered by the possibility of off-target effects of the guide RNAs used here. There are recent examples in which animals have been able to remove disrupted exons, most likely by posttranscriptional splicing processes [18, 19] and a similar process could explain why these transcripts and proteins are detectable here.

In any case, the TRPC6 proteins produced in *Trpc6*^del/del^ rats are non-functional in glomerular cells. Note that two of the three ankyrin repeats in the N-terminal are missing, resulting in disruption of the entire ankyrin repeat domain. Those ankyrin repeats are conserved in all TRPC channels and are necessary for assembly of channels into functional tetramers [27–29]. As an aside, we should note that macroscopic currents through wild-type TRPC6 channels in podocytes [14, 20, 30] do not exhibit dual rectification (at both negative and positive membrane potentials) that is often seen with TRPC6 channels examined in heterologous expression systems such as HEK293 cells [31]. However, dual rectification is not always seen even with heterologously expressed TRPC6 channels [32, 33]. This is by no means unique to podocytes and has been seen in TRPC6 currents in several other preparations [34–38]. The biophysical and biochemical bases for the complex rectifications that are sometimes seen with TRPC6 channels is not known, but it bears noting that this behavior is not captured in barrier-and-well models that recapitulate other complex features of monovalent and divalent cation permeation through TRPC6 [38]. The shape of the I-V curve, beyond some degree of outward rectification at positive membrane potentials, should not be used as a criterion for determining if a particular current is flowing through TRPC6. However, pharmacological criteria (e.g., inhibition by SAR7334) and molecular criteria such as effects of knockdown [20] or knockout [36, 37], or empty vector controls in heterologous expression systems, are more definitive. In this regard, ATP-activated currents in podocytes are also eliminated after TRPC6 knockdown [21].

The core observation in this study is that *Trpc6*^del/del^ rats had less severe kidney disease than *Trpc6*^wt/wt^ controls during the chronic phase when FSGS lesions are present. By contrast, we did not see a protective effect during the acute phase of PAN nephrosis. The acute stage of PAN nephrosis is caused by severe oxidative stress resulting from the metabolism of PAN within podocytes, and it bears noting that the lesions typical of FSGS are not present during the acute stage, even though albuminuria is severe [1, 25, 26].

The effect of the deletion in *Trpc6*^del/del^ in rats, as in *Trpc6*^−/−^ mice studied previously, is probably global. Therefore, there is no reason to attribute all of the protective effects to changes the TRPC6 channels in podocytes, because these channels are expressed in other cell types, including glomerular mesangial cells [11, 39, 40]. Indeed, it is possible that a portion of the protective effects observed in *Trpc6*^del/del^ rats reflects at least mild immunosuppression, as is seen in reduced inflammation associated with airway allergic responses in *Trpc6*^−/−^ mice [41]. TRPC6 channels contribute to Ca^2+^ responses in macrophages [37], T cells [42], and neutrophils [43] and may also play a role in allowing trans-epithelial migration of leukocytes [44, 45]. TRPC6 channels also contribute to fibroblast activation in kidney following unilateral ureteral obstruction in mice [46, 47]. This could explain in part why the reduction of fibrosis in our model was particularly marked. We observed reduced numbers of CD68-positive monocytes and/or macrophages within the glomeruli of PAN-treated *Trpc6*^del/del^ rats, which might occur if TRPC6 was required for their full activation, migration, or secretion of immune modulators.

While we cannot exclude a role for TRPC6 in immunomodulation in PAN nephrosis, FSGS is generally considered to be a podocyte disease, and there is a report that podocyte-specific over-expression of TRPC6 results in glomerular disease in mice [16]. In the rat model studied here, following a loss of a threshold number of podocytes during the acute phase of PAN nephrosis [1], podocytes on the remaining capillary tufts are subjected to increased hydrostatic pressures that can drive their detachment [2, 3, 26]. We have previously observed that TRPC6 becomes active in response to mechanical stimuli in rat podocytes [13, 20]. It is possible that shear and expansile forces associated with glomerular hyperfiltration lead to sustained and excessive activation of TRPC6 in glomerular cells resulting Ca^2+^ overload in foot processes and changes in gene expression, including upregulation of TRPC6 itself [48]. While such changes may be adaptive in the short term, it is possible that sustained activation of TRPC6 eventually increases the loss of podocytes.

The protection seen in *Trpc6*^del/del^ rats, while statistically and biologically robust, was not complete in the later chronic phase of PAN nephrosis (60 days). Clearly, there are multiple processes involved in the pathogenesis of chronic kidney disease and it may be unrealistic to expect complete protection to result from any single manipulation. It is possible that the effect would be more complete if other TRPC channels, such as TRPC5 and/or TRPC3, were also suppressed. TRPC5 is expressed in podocytes [13, 49, 50] and has been suggested to play a role in nephrotic syndromes [51, 52] and can be mobilized to surface under in vitro conditions designed to mimic severe primary FSGS [13]. While we observed that TRPC5 abundance was not altered in the chronic PAN model or in *Trpc6*^del/del^ rats, it is possible that TRPC5 is more important in other forms of kidney disease or in other experimental models. TRPC3 was more abundant in renal cortex of *Trpc6*^del/del^ animals compared to saline-treated *Trpc6*^wt/wt^ littermates. Chronic PAN nephrosis also led to an increase of TRPC3 abundance in *Trpc6*^wt/wt^ animals. However, renal cortical TRPC3 did not increase further following PAN treatment in *Trpc6*^del/del^ rats (Supplemental Fig. 5).

In summary, we have CRISPR/Cas9 methods to delete a portion of the *Trpc6* gene in rats, resulting in a strongly hypomorphic allele that is missing portions of the channel thought to be required for assembly, and which appears to be non-functional. Animals homozygous for that deletion had less kidney disease in the chronic PAN nephrosis model as assessed by multiple measures. These observations have been made in a different species than has been used in previous studies on TRPC6 and in a model that produces severe disease that closely resembles that seen in humans. These observations support therapeutic strategies based on inhibition of TRPC6 channels or targeting the pathways that lead to their dysregulation.

## Electronic supplementary material

ESM 1 (PDF 802 kb)
