# Supplementary material for: Trpc6 inactivation confers protection in a model of severe nephrosis in rats
Source: J Mol Med (Berl). 2018 May 22;96(7):631–44. doi: 10.1007/s00109-018-1648-3 (PMC6015123; doi:10.1007/s00109-018-1648-3)
Supplement: Supplementary file 1 — (PDF 802 kb) [file 109_2018_1648_MOESM1_ESM.pdf]

Trpc6 inactivation confers protection in a model of severe nephrosis in rats.

EUN YOUNG KIM, PARISA YAZDIZADEH SHOTORBANI, STUART E. DRYER

Supplemental Materials

Supplemental Methods for CRISPR/Cas9: The guide RNAs in this study were designed to be specific for TRPC6. Thus, guide RNA 2.1 has 7-bp mismatch with Trpc3, an 8-bp mismatch with Trpc7, and a 7-bp mismatch with Trpc5. Guide RNA 2.2 has a 7-bp mismatch with Trpc3, a 3-bp mismatch with Trpc7 (and there is no protospacer adjacent motif in that region of Trpc7) and a 7-bp mismatch with Trpc5. Pronuclear injections of guide RNAs and Cas9 mRNA were performed on 104 Sprague-Dawley embryos, of which 64 were transferred, and 30 were live-born. These were screened for deletions by PCR using standard methods (see further below), and genomic DNA from five founder animals with deletions of the predicted size was sequenced. These founder animals had deletions within Exon 2 that resulted in frame-shifts that introduce numerous stop codons downstream of the deletion. Exon 2 of *Trpc6* encodes a substantial portion of the ankyrin-repeat domain in the N-terminal of the TRPC6 channel protein, upstream of the first transmembrane domain. Two of the founders were chosen for breeding and transmission of the knockout allele was confirmed by sequencing and PCR analysis. These animals were used to expand a *Trpc6*$^{del/del}$ colony in our animal facility.

Table 1. Sequences of primers used for genotyping and for analysis of *Trpc6* transcripts.

**Primer pairs for genotyping**	
Trpc6 forward:	AATGGCAGGTCATTTAGCATACG
Trpc6 reverse:	ATCTTGCTGGAGTTCAGACTGG
**Primer pairs for RT-PCR**	
Exon 1 forward:	CGAGAGCCAGGACTATCTGC
Exon 2 reverse:	TTGTTTCTGGCTGCATTCTG
Exon 2 forward:	CAGAATGCAGCCAGAAACAA
Exon 4 reverse:	CTGCAAGGAGCACACCAGTA
Exon 4 forward:	TGAAATTCCTCGTGGTCCTC
Exon 8 reverse:	TGACCACCGACTTCACTTCA
Exon 8 forward:	TGAAGTGAAGTCGGTGGTCA
Exon 13 reverse:	TCCCCTTCGTTCACTTCATC
Exon 1 forward:	CTATCTGCTGATGGAC AGC
Exon Δ2 reverse:	GAAAGCGTTCCTCCTCAATAGAC
Actb forward:	AGCCATGTACGTAGCCATCC
Actb reverse:	CTCTCAGCTGTGGTGGTGAA

The PCR cycling protocol used for RT-PCR was: 94° for 5 min, and 16 cycles of 94° for 30 sec, 55° for 30 sec, 72° for 1 min: and a final extension at 72° for 5 min.

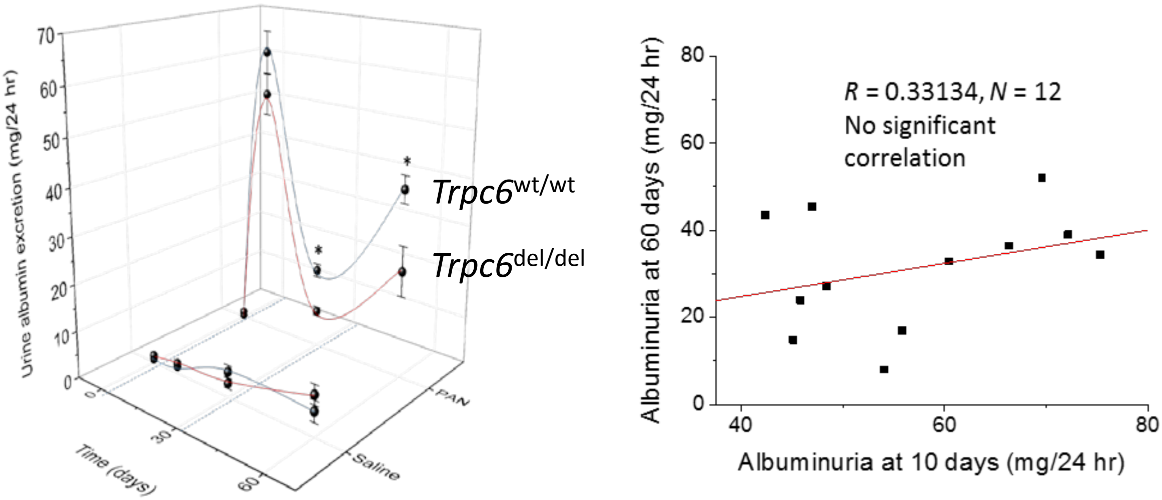

Supplemental Figure 1. Graphical summary of experiments in Fig. 2 (left), and regression analysis showing that the magnitude of albuminuria at the 60-day time point, which is after the second PAN injection, is not correlated with the degree of albuminuria seen during the acute phase, 10 days after the first PAN injection (right).

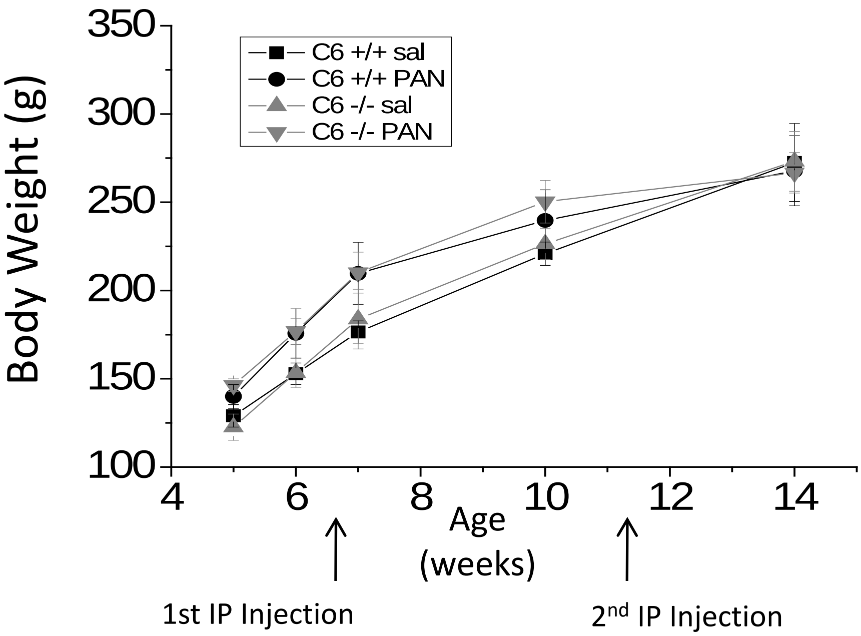

Supplemental Figure 2. Rats during acute PAN nephrosis are slightly heavier than saline-treated controls, regardless of genotype, possibly due to edema during the acute phase. By the time the animals were sacrificed, there were no differences in weight on the basis of drug treatment group or genotype.

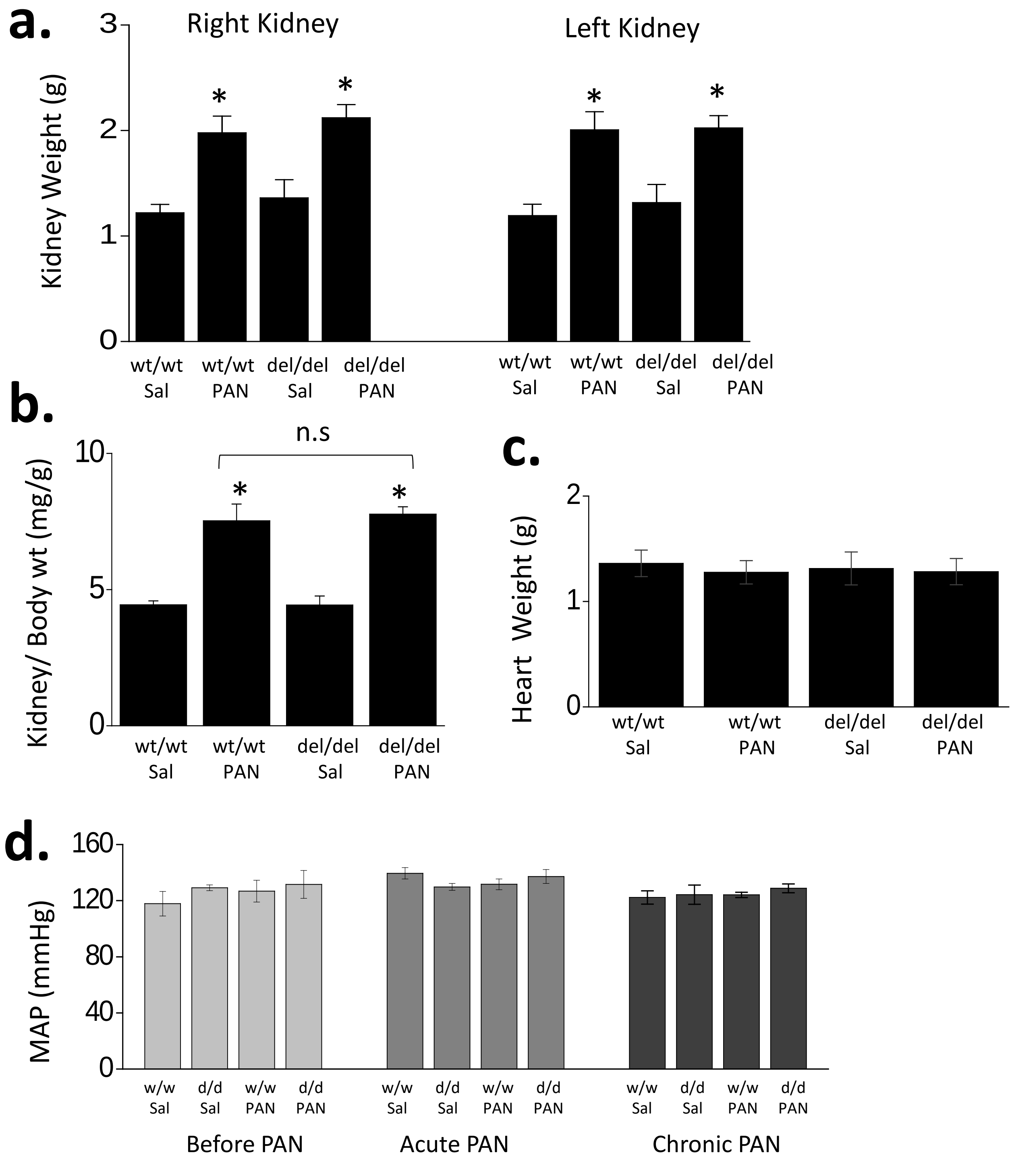

Supplemental Figure 3. Kidney weights and kidney weight/body weight ratios, heart weight, and mean arterial pressure (MAP) for the same animals shown in Fig. 2 at the time of sacrifice. Note that Exon 2 deletion did not affect the increases in kidney weight/body weight ratio in PAN-treated rats. PAN treatment and genotype had no effect on heart weight or MAP..

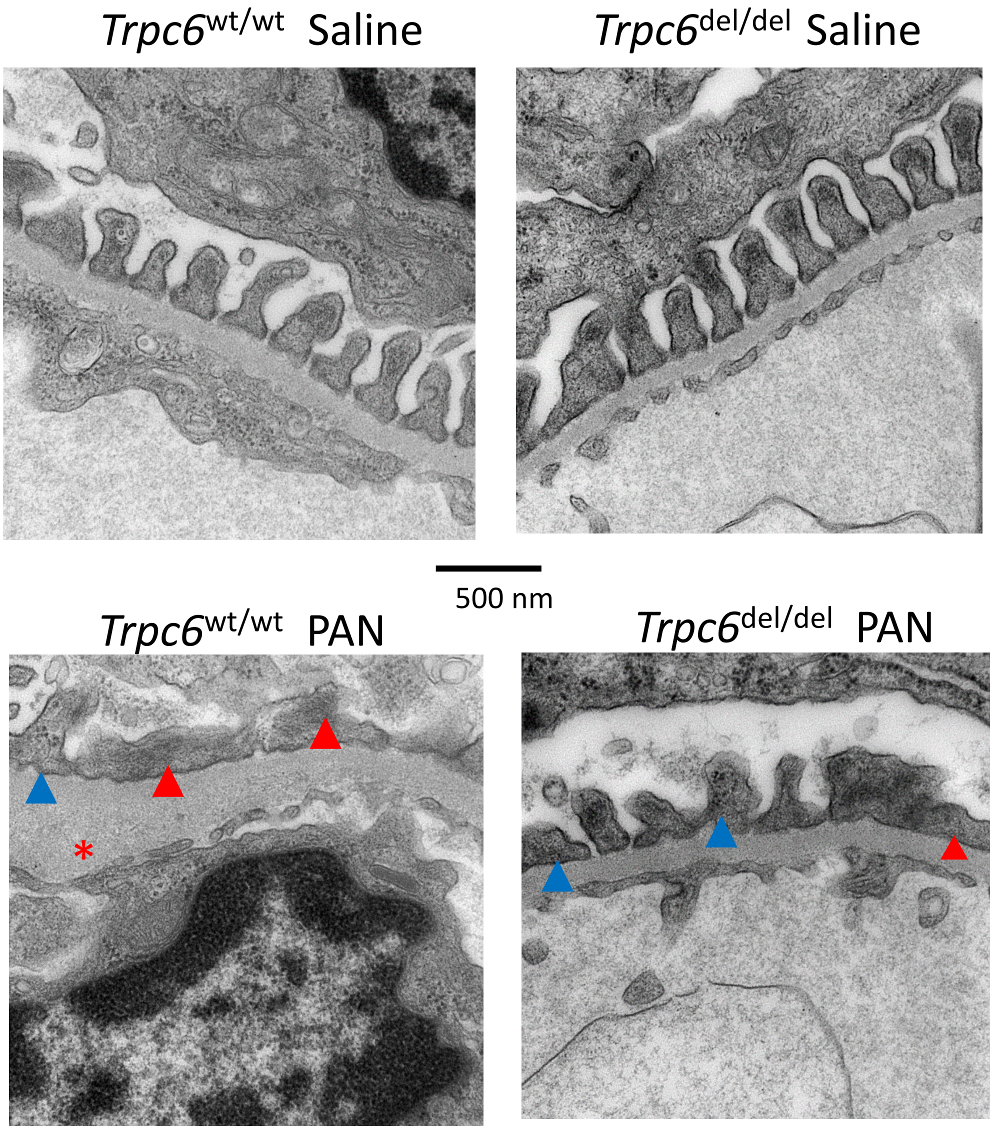

Supplemental Figure 4. Higher magnification images of scanning electron microscopy showing foot process and glomerular basement membrane morphology in control animals and during chronic PAN nephrosis as indicated. Red arrow heads show regions in which foot processes are not present, and blue arrow heads indicate abnormally wide foot process. Red asterisks indicates thickened basement membrane.

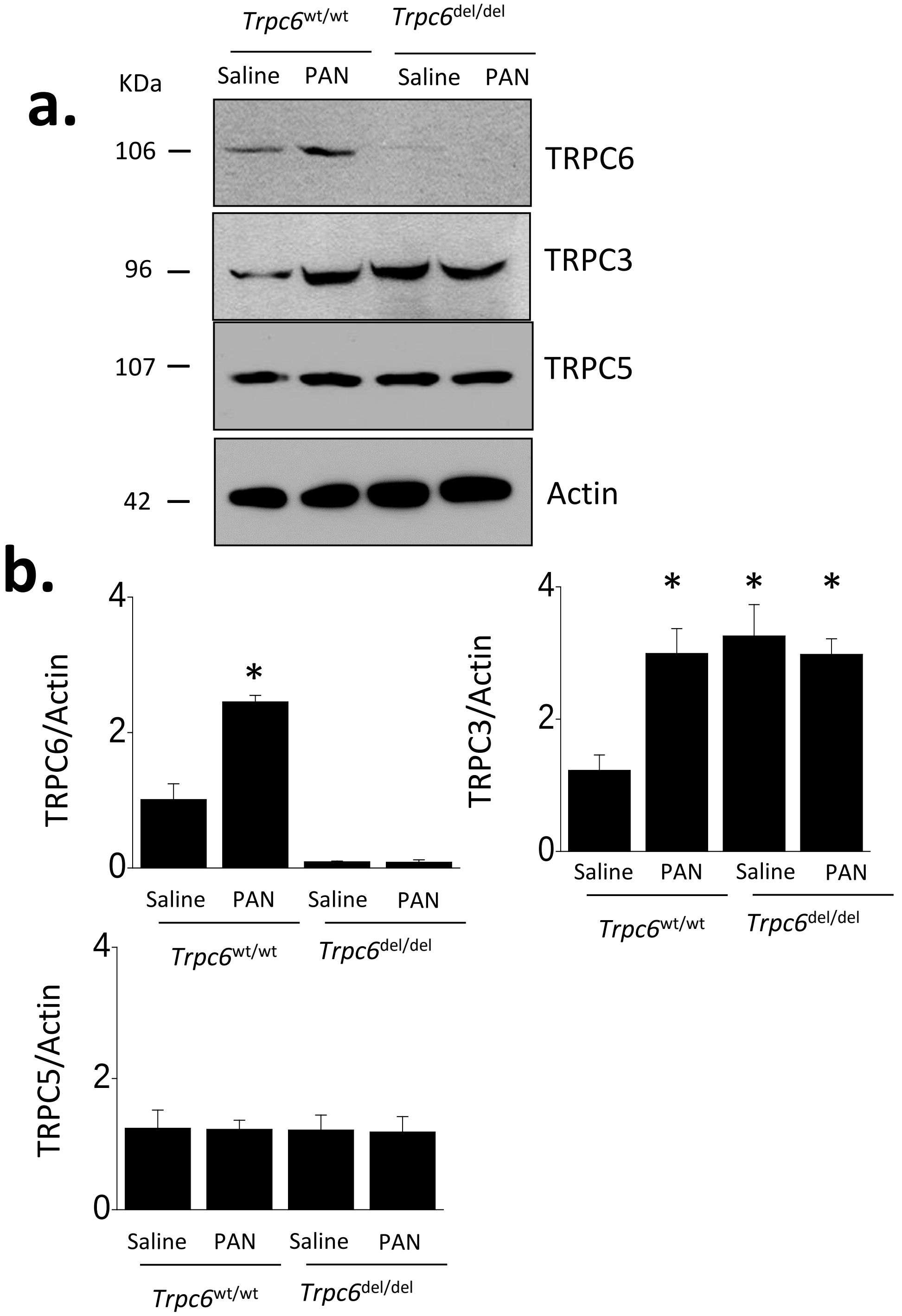

Supplemental Figure 5. Renal cortical expression of TRPC channels. (A) Representative immunoblot analysis (one rat per lane). In *Trpc6*$^{wt/wt}$ rats, chronic PAN causes marked increases in the overall abundance of TRPC6 and TRPC3, but has no effect on TRPC5. Note the extremely faint TRPC6 signal in the *Trpc6*$^{del/del}$ rats. TRPC5 is not different in any group. TRPC3 is increased in PAN nephrosis in wild-type rats but does not increase further above this in chronic PAN *Trpc6*$^{del/del}$ rats. (B) Summary of densitometric analysis of immunoblots from all of the animals initially shown in Fig. 3, with signals of the appropriate molecular weight normalized to actin.
